# Pitcher's disease: A case of brain fog, arm weakness, numbness, and tingling

**DOI:** 10.1016/j.jvscit.2026.102414

**Published:** 2026-07-14

**Authors:** Alec Jotte, Nicole Kilada, Michele Richard, Edward Qian

**Affiliations:** a Department of Internal Medicine, Rush University Medical Center, Chicago, IL; b Rush Medical College, Rush University, Chicago, IL; c Department of Vascular Surgery, Rush University Medical Center, Chicago, IL

**Keywords:** Thoracic outlet syndrome, Neurovascular compression, Polyneuropathy, Brain fog, Case study

## Abstract

Thoracic outlet syndrome (TOS) is a rare condition caused by compression of the lower trunk of the brachial plexus and/or subclavian vessels. This case report describes the presentation of a 56-year-old right-handed baseball pitcher who presented with 1 year of right arm pain, finger tingling, heaviness, and brain fog. A vascular duplex ultrasound guidance and functional maneuvers showed evidence of venous and neurogenic TOS. The patient was successfully treated with a first rib resection, substantially improving right arm symptoms and brain fog. This case highlights a correlation between nonspecific cognitive dysfunction and TOS, which is seldom reported in existing literature.

Thoracic outlet syndrome (TOS) is a rare condition (1-3 per 100,000) caused by compression of the lower trunk of the brachial plexus and/or the subclavian vessels.^1^ It is a heterogenous entity defined by symptoms caused by vascular or neurogenic compression in the thoracic outlet, an anatomical region including the interscalene triangle, costoclavicular space, and subcoracoid space.^1^ TOS can be neurogenic (TOS, 95% of cases), venous (TOS, 4% of cases), or arterial (TOS, 1% of cases).^1^, ^2^, ^3^, ^4^ TOS has notoriously ambiguous diagnostic criteria, and remains largely a clinical diagnosis of exclusion, though imaging such as magnetic resonance imaging (MRI), computed tomography, and duplex Doppler ultrasound study can aid in diagnosis.^1^^,^^5^^,^^6^ This retrospective case study explores the presentation of a 56-year-old right-handed baseball pitcher who presented with 1 year of nonspecific cognitive symptoms in addition to right arm pain, finger tingling, and heaviness. The patient has consented to publication of case details and images.

## Description

**Fig 1:**
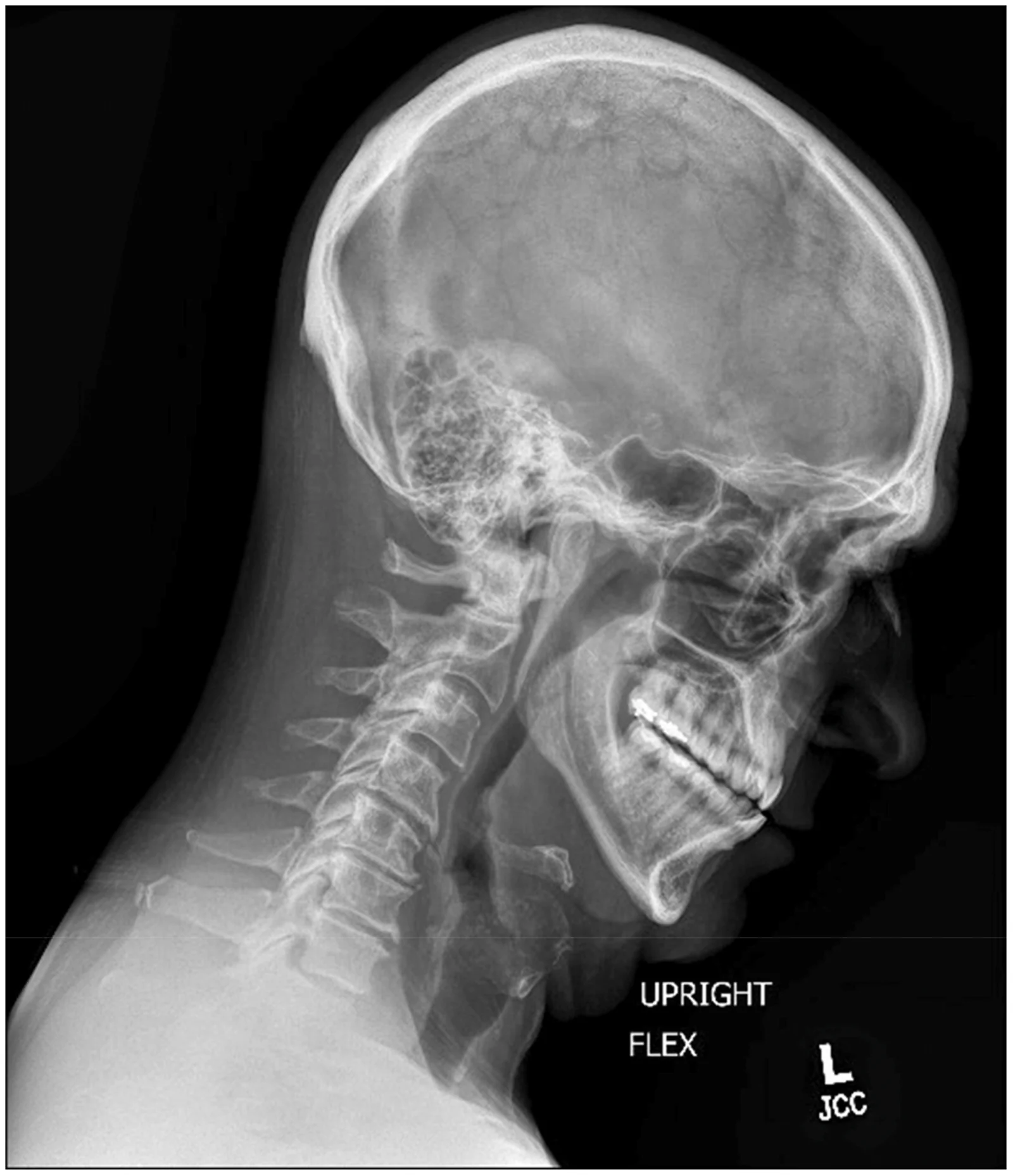
Multilevel facet degenerative changes, reduced disk height at C3 to C4 and C5 to C6. Absence of a cervical rib.

**Fig 2:**
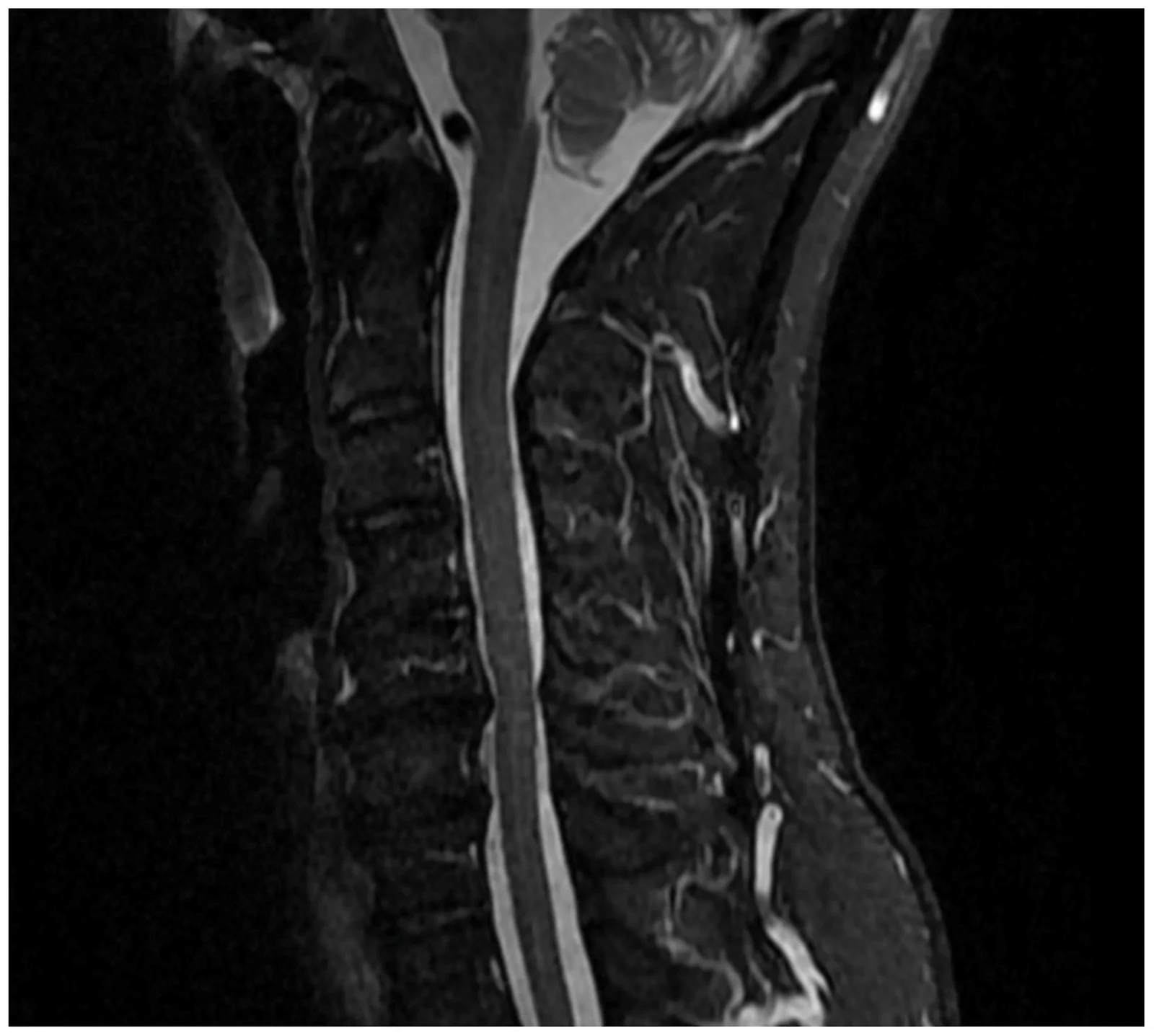
T2 sequence. Degeneration of disk and loss of height at the C5 to C6 level. Diffuse narrowing of thecal sac consistent with spinal stenosis.

**Fig 3:**
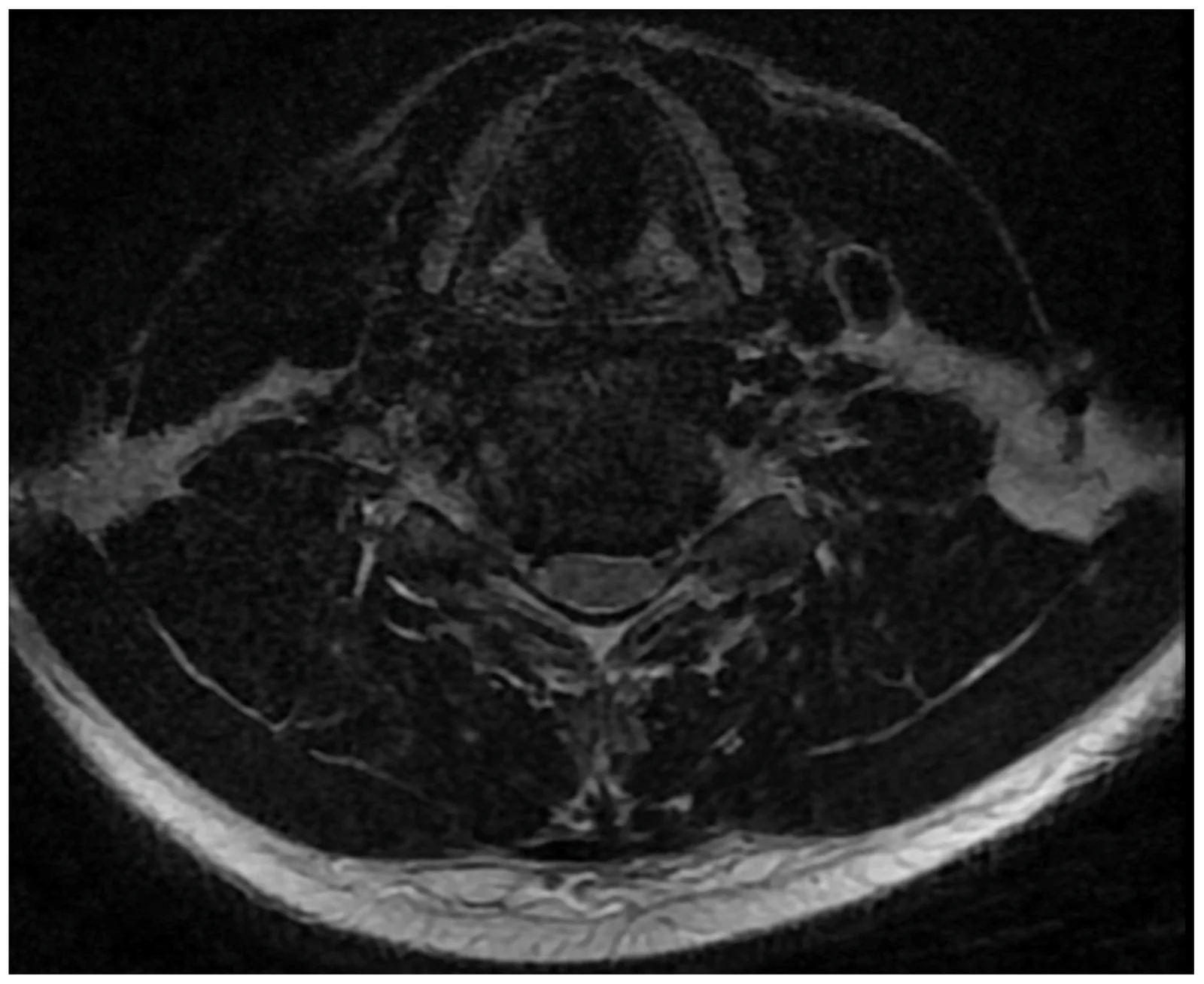
T2 sequence. Degeneration of disk and loss of height at the C5 to C6 level. Diffuse narrowing of thecal sac consistent with spinal stenosis.

A 56-year-old right-handed lifelong recreational baseball pitcher with no significant past medical history presented for evaluation of 1 year of right arm pain, finger tingling, heaviness, and brain fog. Previous evaluations by neurology and neurosurgery revealed a mild sensory polyneuropathy and degenerative changes seen at the C5 to C6 level on MRI and the absence of a cervical rib (Fig 1, Fig 2, Fig 3). He completed a course of physical therapy (PT) with mild improvement, though he noted full return of symptoms. He also concomitantly saw rheumatology where he had autoimmune laboratory work done that was unrevealing. Given continued symptoms, his primary care physician referred him to Sports Medicine for further evaluation. In clinic, the patient reported sudden onset pain and numbness going down his right arm with “loss of power” and associated “brain fog,” which he described as feeling sluggish with low energy, forgetfulness, and having difficulty concentrating. There was no preceding trauma. He reported his pitch count had decreased to where he could only throw 30 pitches before his symptoms became unbearable.

Physical examination revealed a well appearing man in no acute distress, resting comfortably on the examination table. Examination of the right and left shoulders revealed no redness, warmth, swelling, or tenderness to palpation. No prominent venous collaterals were observed on the shoulder or chest. Bilateral shoulder range of motion revealed full-active flexion, extension, abduction and adduction, and internal and external rotation. Strength was 5/5 in bilateral upper extremities throughout. Bilateral Adson tests were negative. Spurling compression tests were negative bilaterally. Neer test, empty can, and shoulder drop tests were negative. Scapular assistance test was negative bilaterally. Roo test was positive on the right side.

Given the patient’s history and physical examination, the differential included TOS (vascular vs neurogenic), cervical radiculopathy (C5-C6 stenosis) secondary to degenerative cervical disk disease, peripheral nerve entrapment (suprascapular, axillary, long thoracic nerve, and radial nerve), brachial plexopathy (Parsonage-Turner syndrome), inflammatory/autoimmune neuropathy, mononeuritis simplex/multiplex, superior labrum anterior to posterior lesion, and a biceps tendon tear.

An electromyography revealed a moderately reduced amplitude with borderline slow conduction velocities of the right radial nerve. Needle examination of the deltoid, biceps, and triceps and right paraspinals muscles (C5/C6, L5/S1) were normal. Results were consistent with a mild sensory axonal polyneuropathy with no evidence for right cervical radiculopathies. MRI of the brain and neck failed to reveal any neurological explanation for the patient’s brain fog. MRI revealed no evidence of intracranial hemorrhage or fluid collections and no areas of ischemia.

**Fig 4:**
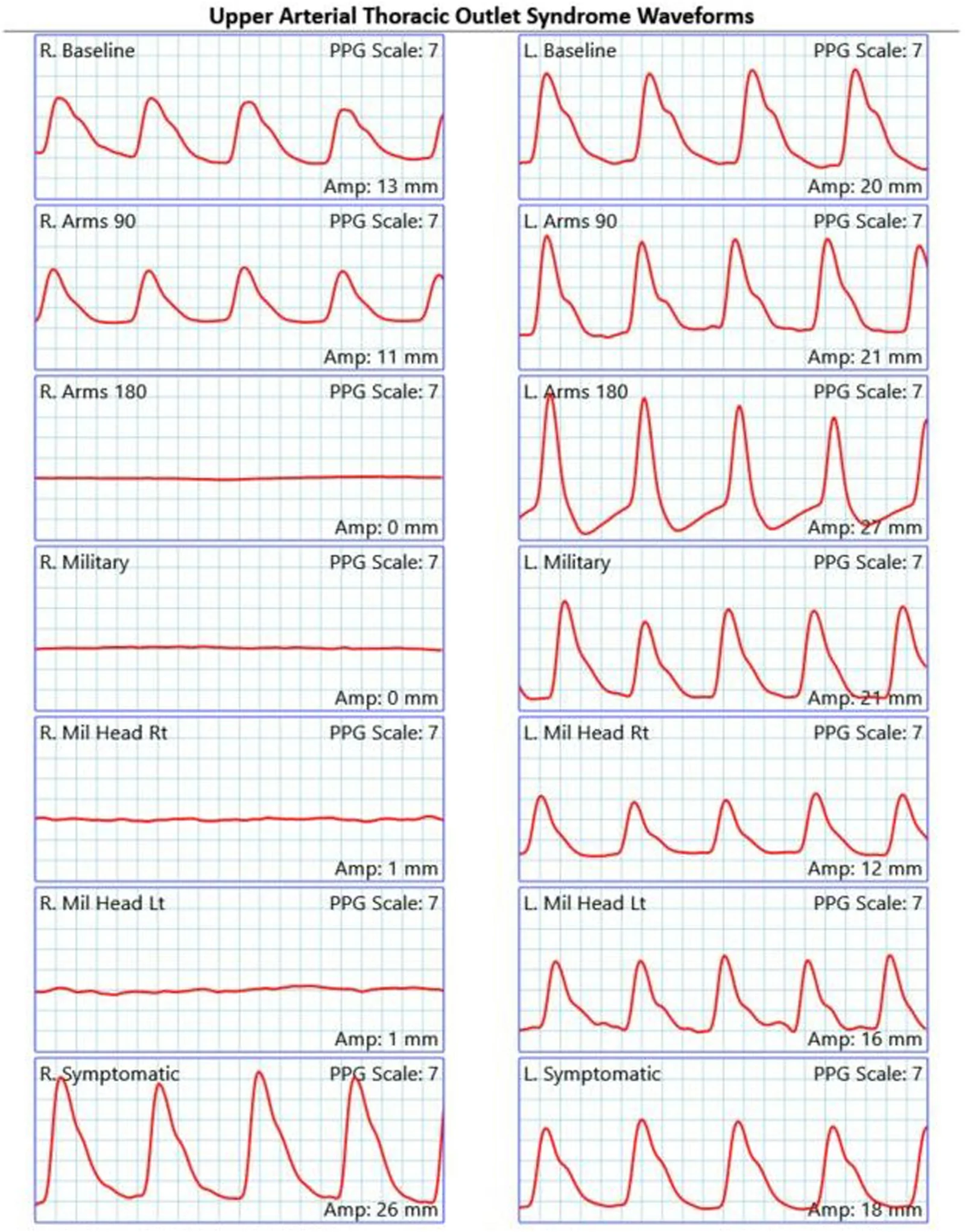
Continuous wave Doppler via photoplethysmography of the bilateral upper extremities with the arms in multiple positions. Evidence of dynamic right arterial obstruction with provocative movements.

Vascular duplex ultrasound guidance of the upper-extremity veins revealed decreased color Doppler signal and flow within the distal right subclavian vein, with rouleaux flow, with the patient’s arm at 90°. Upper-extremity arterial duplex ultrasound guidance and functional maneuvers showed evidence of TOS involving the right upper extremity at 180° and military position with head to the right and left (Fig 4). The right wrist-brachial index also demonstrated compression. No evidence of TOS involving the left upper extremity was observed. The final working diagnosis was right-sided venous and neurogenic TOS.

The patient was referred to vascular surgery who performed a scalene block with improvement of pain for 2 to 3 days, further confirming the likelihood of TOS. The patient went on to have a first rib resection. Two weeks postoperatively, the patient reported his brain fog resolved, and his arm felt much better. Three months postoperatively, the patient reports continued substantial improvement in right arm symptoms and brain fog, and he has returned to his previous level of pitching performance. A 12-month postoperative follow-up revealed no return of symptoms.

## Discussion

Given that TOS is a rare condition (1-3 in 100,000) and a clinical diagnosis of exclusion, identifying cases with heterogenous, nonspecific symptoms can prove challenging. The diagnosis is further complicated by substantial symptom overlap with similar and commonly comorbid conditions such as cervical radiculopathy,^2^, ^3^, ^4^ as was seen in our patient. Patients who perform repeated arm and shoulder movements through work, weightlifting, and athletics are at an increased risk for developing TOS due to hypertrophy of the scalene muscles,^1^, ^2^, ^3^, ^4^ and baseball pitchers have been a population uniquely identified as high risk for the development of TOS.^1^^,^^4^^,^^5^^,^^7^

Nonoperative management with PT focusing on reducing tensile or compressive loads across the thoracic outlet region and behavioral modifications are the initial therapy of choice^1^, ^2^, ^3^, ^4^^,^^7^^,^^8^ and is successful in up to 70% of patients with neurogenic TOS.^7^^,^^8^ Should conservative therapy fail, a local injection of botulinum toxin, local anesthetic, or steroids into the anterior scalene are used.^4^ Relief of symptoms, particularly by lidocaine block, predicts the likelihood of successful resolution of symptoms following surgical intervention.^1^^,^^2^^,^^4^^,^^9^^,^^10^ Surgical interventions include surgical resection of the first rib or the scalene muscles,^1^, ^2^, ^3^^,^^7^^,^^8^^,^^11^^,^^12^ and studies have shown success rates as high as 90% in symptomatic improvement following surgery.^12^^,^^13^ Smaller studies have evaluated the roles of PT, botulinum injections, and surgery in collegiate and professional athletes.^5^^,^^14^ Most experience relief of symptoms and return to play with conservative measures, and those who do require surgery have excellent outcomes with nearly all returning to play within 1 year.^15^, ^16^, ^17^

Brain fog as a possible symptom of TOS had not appeared in the literature before 2025 when a case report was published highlighting the resolution of long standing, nonspecific cognitive “fog” following surgical correction of TOS in a patient in Maryland.^18^ Similarly, our patient experienced rapid resolution of his persistent brain fog following surgical correction of his TOS. It is known that cervical degenerative disease and sleep disturbance are related,^19^ though our report highlights that there may be a link between thoracic outlet compression and nonspecific cognitive dysfunction. Yin et al^19^ proposed that the neurovascular compression and chronic painful stimuli in TOS could contribute to increased sympathetic reactivity and increased intercranial pressure both leading to poor sleep and autonomic dysfunction. This pathophysiology could be responsible for the clinical symptoms of “brain fog,” although a direct causal mechanism remains uncertain. Additional research is required to better understand this phenomenon.

## Conclusions

TOS is a rare condition and often a clinical diagnosis of exclusion in which patients can present with a variety of symptoms depending on the nature of the vascular and/or neurogenic compression present. This case of a 56-year-old pitcher highlights an association between TOS and nonspecific cognitive dysfunction, or “brain fog,” which requires additional research to further elucidate.

## Funding

None.

## Disclosures

None.
